# Supplementary figures and images for: Cerebellar Golgi cell models predict dendritic processing and mechanisms of synaptic plasticity
Source: PLoS Comput Biol. 2020 Dec 30;16(12):e1007937. doi: 10.1371/journal.pcbi.1007937 (PMC7837495; doi:10.1371/journal.pcbi.1007937)

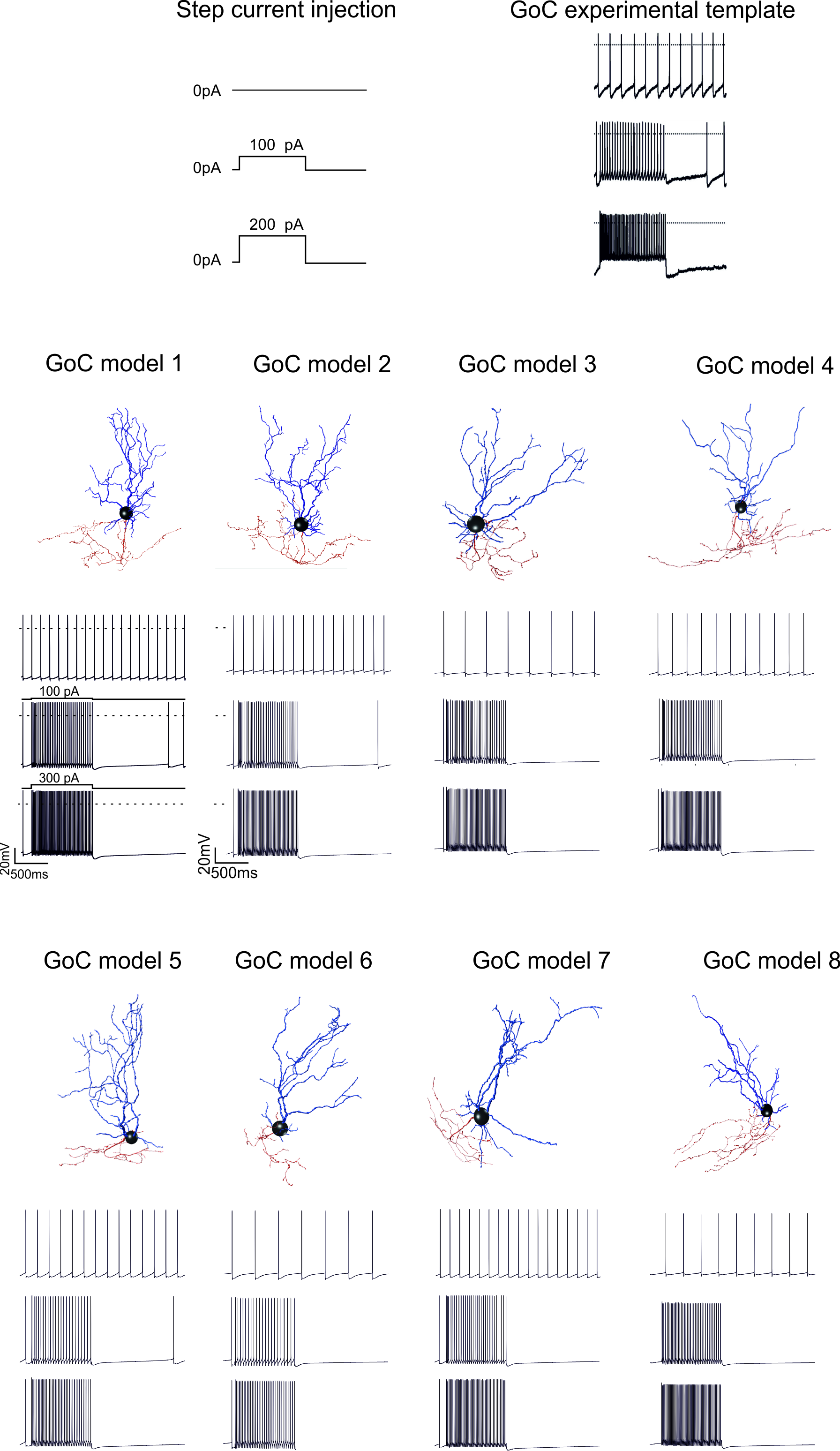

Supplement: S1 Fig — The GoC experimental template is shown to the top along with the step current injection protocol used to elicit the electrical response [15]. The same stimulation protocol was applied to the 8 Golgi cell models. For each model the panels show the morphological reconstruction and the electrical response. Note the similarity among the models and between them and the experimental case. (TIF) [file pcbi.1007937.s003.tif]

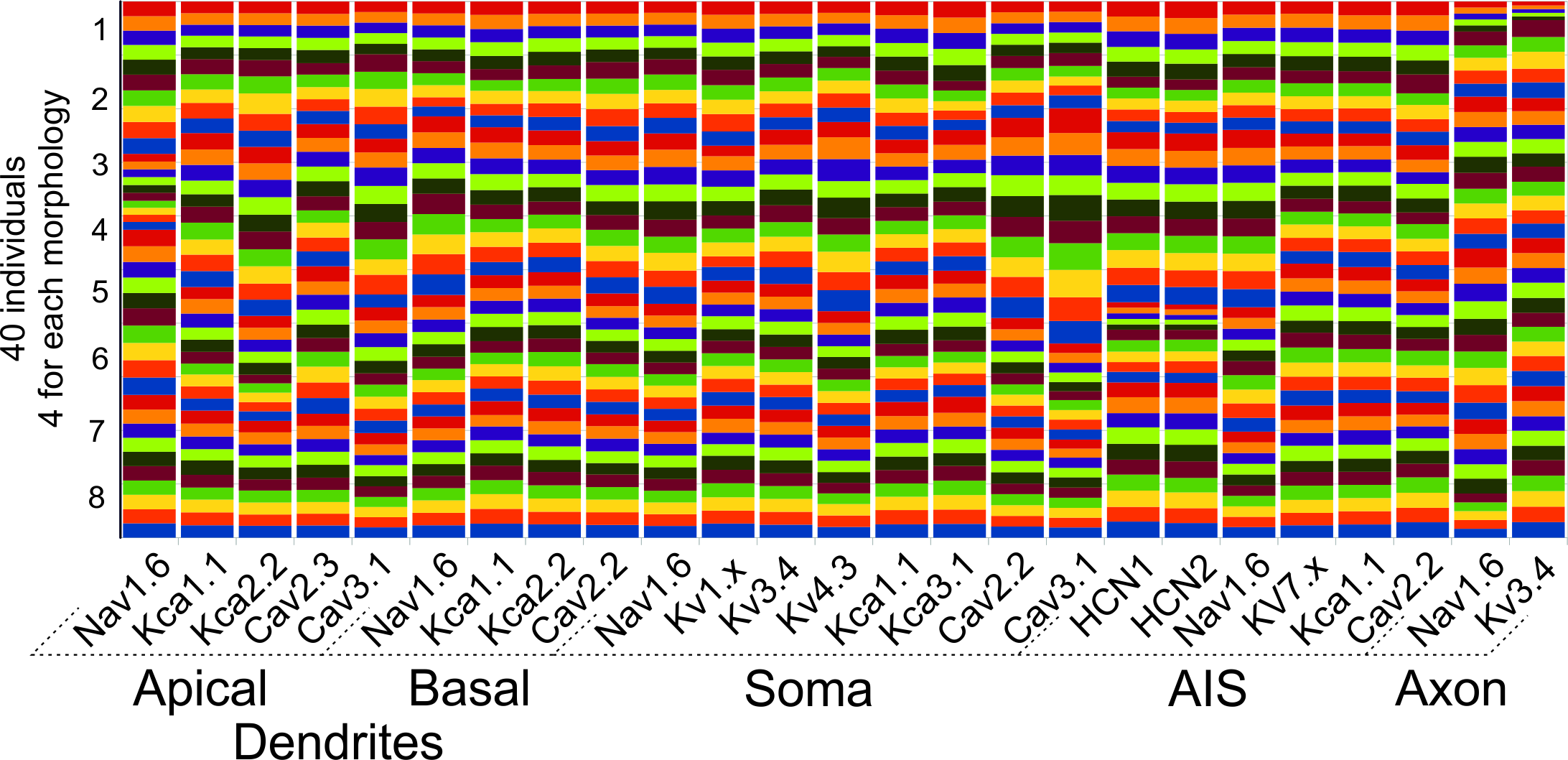

Supplement: S2 Fig — The table shows the maximum ionic channel conductances of 5 randomly chosen individuals for each of the 8 GoC models. The 40 individuals (all validated according to the criteria explained in Materials and Methods) show that the model optimization algorithms thoroughly explored the parameter space providing diverse ionic conductance patterns. In other words, there are different combinations of conductance values that allow to achieve a spike discharge compatible with the experimental templates. (TIF) [file pcbi.1007937.s004.tif]

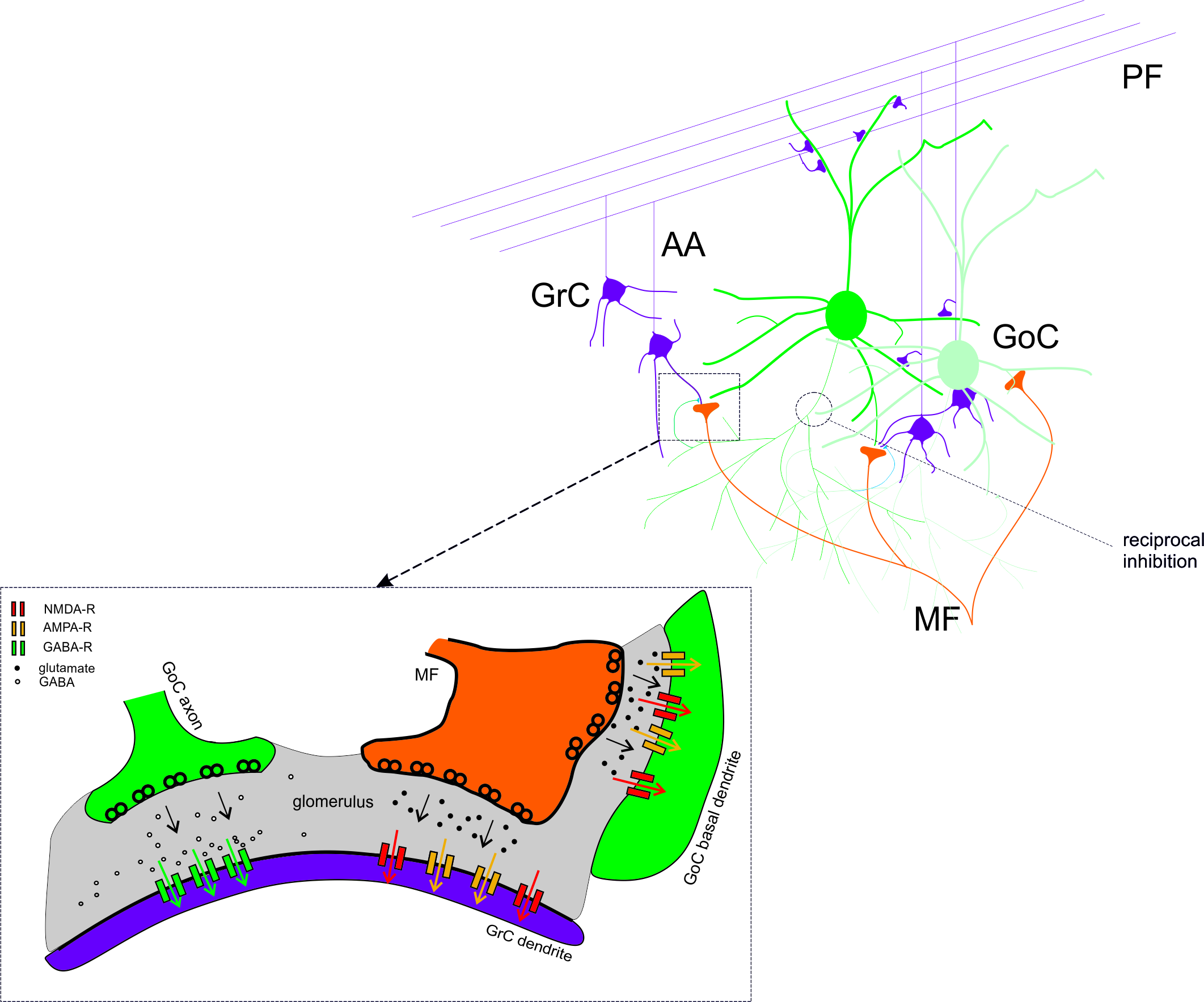

Supplement: S3 Fig — The figure illustrates the connectivity of GoCs with MFs and GrCs. Inside the glomerulus, a Golgi cell basal dendrite receives excitatory inputs from a MF terminal, while the Golgi cell axon inhibits granule cell dendrites. The granule cells excite the Golgi cell dendrites thought the AAs and PFs. The PF activate only AMPA receptors, while the AA and MF activate both AMPA and NMDA—NR2B-containing receptors. Reciprocal inhibition occurs between the GoC axons and dendrites. Golgi cell; GrC, granule cell; AA, ascending axon; PF, parallel fiber; MF, mossy fiber. (TIF) [file pcbi.1007937.s005.tif]

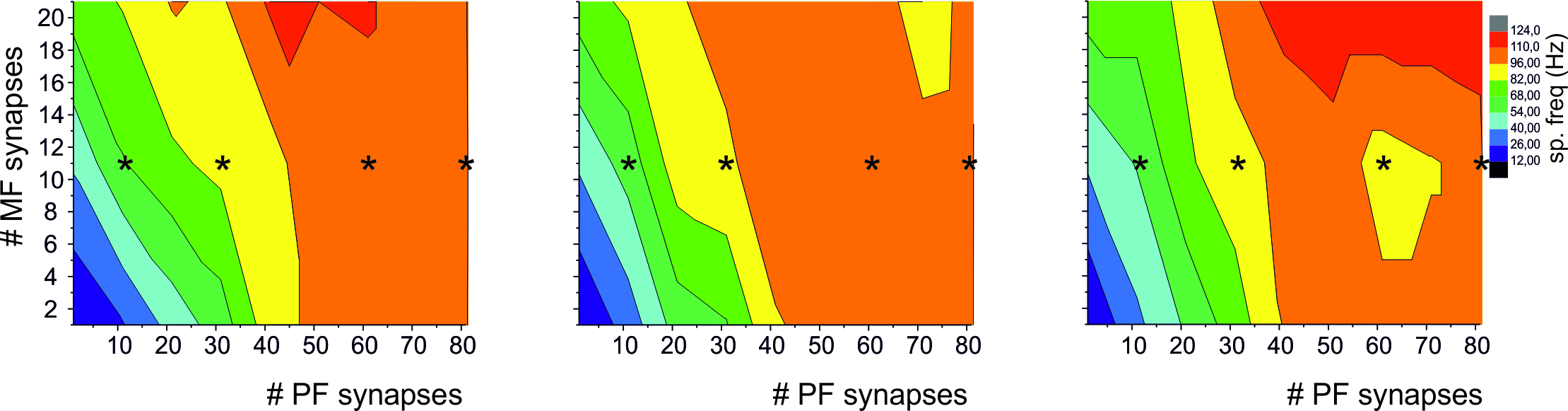

Supplement: S4 Fig — The synaptic input-output (I/O) space of the Golgi cell model was computed from the response to combined activation of basal and apical dendrites through the corresponding input pathways. The simulations have been repeated using a short train of 5spikes@100Hz on both mossy fibers and parallel fibers and the corresponding output frequency is color-codeded. The stars indicate 4 points in the I/O plane that were used to compute STDP, as shown in S5 Fig. (TIF) [file pcbi.1007937.s006.tif]

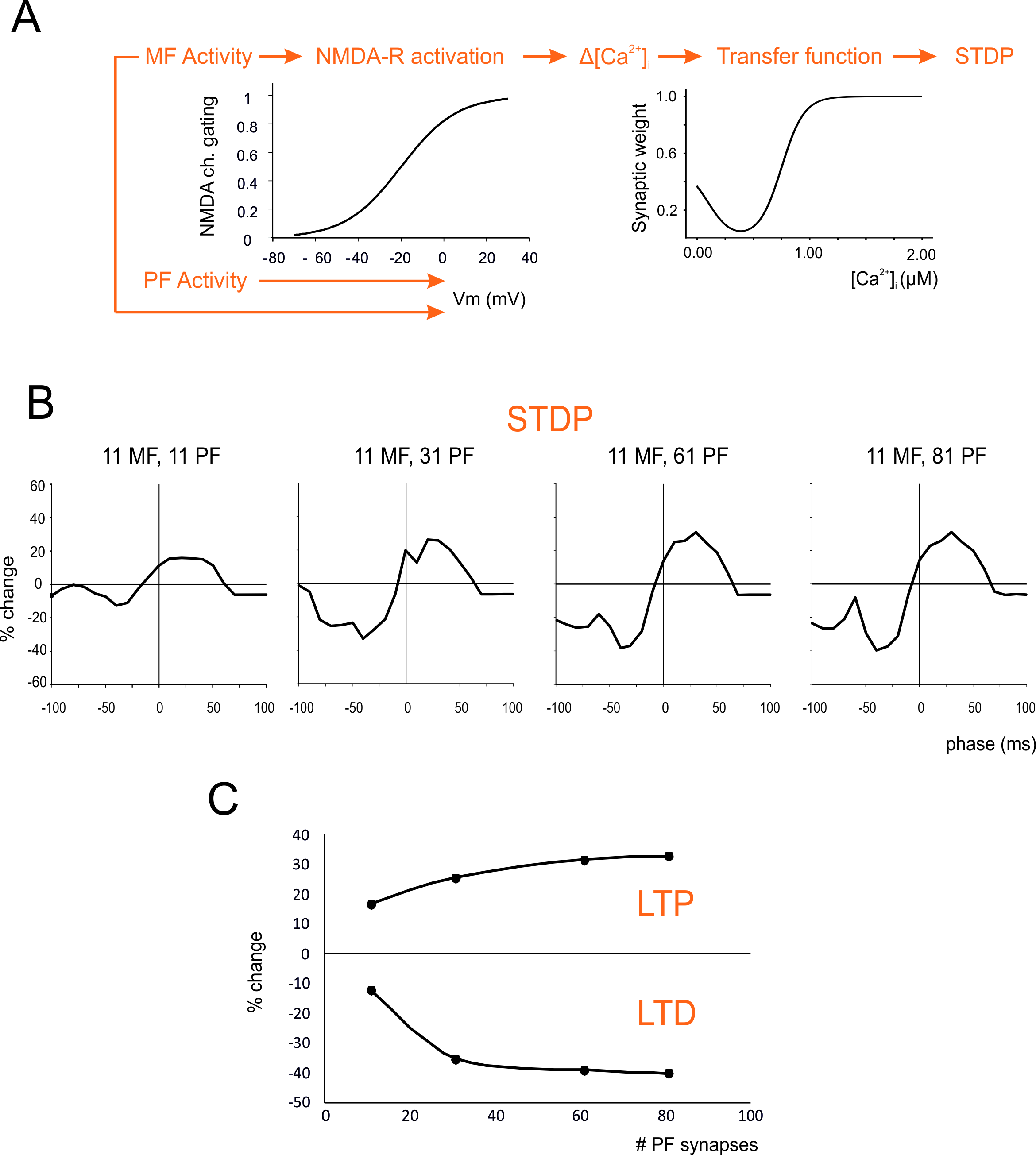

Supplement: S5 Fig — (A) Schematics of the STDP model applied to Golgi cells [42,43]. The coincidence of MF and PF activity regulates NMDA-R activation and channel unblock causing Ca2+ entry and a change in Ca2+ concentration, Δ[Ca2+]i. This, in turn, is transformed into STDP by sigmoidal transfer functions accounting for the molecular mechanisms of Ca-dependent plasticity. (B) Based on the I/O plots of S4 Fig, our simulations used 11 mossy fiber synapse and an increasing number of parallel fiber synapses (11, 31, 61, 81), both stimulated with a 5spikes@100Hz burst. The Δ[Ca2+]i. generated by NMDA channels in the corresponding dendritic compartment reflects local Ca regulation, including removal due to diffusion and extrusion but not amplification by local calcium stores. This amplification, e.g. in the neighboring granule cell dendrites, is of about 3 times [81]. Therefore, the Δ[Ca2+]i. caused by NMDA channel opening was multiplied by 3 times bringing Δ[Ca2+]i. around the STDP transition point (~0.75 μM). With a rate of change of 10, the model yields a classical STDP curve for the Golgi cell with a gain that depends on the amount of depolarization conveyed by the parallel fibers acting on apical dendrites. (C) Dependence of STDP magnitude on the number of active parallel fibers. The gain tends to plateau around ±40%. (TIF) [file pcbi.1007937.s007.tif]
