# Supplementary material for: Cerebellar Golgi cell models predict dendritic processing and mechanisms of synaptic plasticity
Source: PLoS Comput Biol. 2020 Dec 30;16(12):e1007937. doi: 10.1371/journal.pcbi.1007937 (PMC7837495; doi:10.1371/journal.pcbi.1007937)
Supplement: Attachment — Submitted filename: Reviewer_answer_v2.docx [file pcbi.1007937.s011.docx]

Reviewer #1:

The manuscript “Cerebellar Golgi cell models predict dendritic processing and mechanisms of synaptic plasticity” from Masoli et al is a solid description of a new model of GoCs that recapitulate many of the intrinsic properties known to exist in cerebellar GoCs. They use a machine learning method -- ‘genetic optimization’ -- to find channel conductance values that produce known electrophysiological signatures. They go on to do experiments with the model, leaving out various conductances, to identify KCa conductances and a link with Ca2.2 as critical in steady pacemaking, as their removal promotes a bursting phenotype. Synaptic inputs to both apical and basal dendrites elicit a ‘burst’ of spikes and through asymmetry in action potential back propagation speed may support coincidence detection and subsequent spike time dependent plasticity. The manuscript was very clear, well written and may become a definitive model of Golgi cells.

Below I provide minor suggestions for improvement.

Main critiques.

1. The optimization process used trains on known intrinsic properties of Golgi cells. I would imagine that with the number of currents the authors use that multiple solutions are found, producing a solution ‘space’. Rather the findings are more rigid, with one answer per morphology. Can the authors please address this or provide a description of the parameter spaces found that recapitulate known Golgi cell intrinsic properties?

*We thank the reviewer for pointing out the issue. Indeed, each model shown in the paper is selected from a large set of possible models (1152). About 60% of these models were discarded because they did not match the typical features of a Golgi cell (i.e. did not show spontaneous firing, an appropriate Input-Output relationship or a sag). Among the models surviving the first triage, only a part (about 10% of the total) showed correct sensitivity to ionic channel blockers. Out of these about 100 models, one was randomly chosen and shown in the paper. This is now explained in the Methods. Moreover, the parameter space is illustrated in a new figure in Supporting Information.*

2. The model of the synaptic inputs seems rather limited. What are the studies that support the numbers chosen for mossy fibers (20), and parallel fibers (89 synapses)? These seem very specific and not necessarily correct. Relatedly, I found the focus on short-term synaptic plasticity to be interesting but not necessarily complete for the potential roles of Golgi cells in the circuit. The authors might also comment on the ability of the Golgi cells rate to track synaptic inputs to their basal dendrites, apical dendrites or both in ways that could enhance computation within the granule cell layer – possibly expanding the description of data in Figure 7.

*The number of synaptic inputs impinging on Golgi cell dendrites has been estimated in a recent paper (Cesana et al., 2013) yielding about 400 ascending axon synapse, 4000 parallel fiber synapses, and 40 mossy fiber synapse per Golgi cell. It is not the aim of this paper to resolve the full synaptic complement of the cell (we have another manuscript under preparation that is dealing with network reconstruction, in which synapses are located on the dendritic tree depending on specific geometric and intersection rules). Here we have partially explored the synaptic input space in order to generate EPSPs and spikes in Golgi cells and investigate principles of dendritic processing, especially backpropagation and coincidence detection.*

*We thank the reviewer to give us the occasion to comment upon the potential implication of short-term synaptic plasticity at the different Golgi cell synapses. Short-term facilitation at ascending axon and parallel fiber synapses should make the Golgi cell poorly sensitive to sparse low-frequency spikes but very sensitive to spike bursts at more than 10 Hz. In this way, Golgi cells should prevent circuit run-away at high activity regimes. The mossy fiber synapses, which do not have apparent facilitation or depression during bursts, are apparently designed for reliable transmission at all frequencies, providing the basis for a linear input-output transformation.*

*Golgi cell resonance may be able to improve synaptic input tracking in the theta-band, providing further support to plasticity mechanisms operating on this frequency band.*

*These aspects are now mentioned in the Discussion and illustrated in a new figure in supplemental Information.*

3. The authors should mention that the Golgi cell reconstructions from slices are necessarily incomplete, so although these reconstructions are valuable for these purposes they are not as large as Golgi cells actually are.

*We agree with this comment. The available Golgi cell reconstructions have rather complete dendritic trees but are missing an extensive part of the axonal plexus ((Tabuchi, Gilmer, Purba, & Person, 2019).*

4. Ln 318-320, the authors could expand on the observation that inhibition “dumps the burst” and influences LTP. I found the comment terse and could be expanded upon. Do the authors mean “prevents the burst” and if so, analyses on to what extent the spike rate is reduced to prevent LTP are warranted.

*The effect of inhibition is illustrated in Fig. 7. Clearly the effect of inhibition is actually to reduce the burst (the wording has been changed). The burst is prevented only when the number of inhibitory synapses crosses a threshold of about 10 in these simulations. The impact of inhibition on NMDA receptor-dependent long-term synaptic plasticity is illustrated in Fig. 9C. In principle, modulating the burst through inhibition would allow to visit a relevant portion of the curve in fig. 9C and to modify plasticity to a variable extent. The further investigation of this aspect would require suitable induction rules, one of which is now exemplified in Supporting Information.*

Minor

1. The final sentence of the Methods seems to be an incomplete though or just a punctuation typo.

2. I didn’t follow the language in the methods, used twice, that “This allowed to prevent” ln 581 or “This allowed prevent” ln 575. What is “this” and what is allowed to prevent in these sentences? Prevented?

*We corrected these typing errors.*

Reviewer #2:

The article represents a valuable contribution to computational modeling of the cerebellum and proposes an interesting mechanisms of cerebellar plasticity. It is well argued, well written and well researched. The main conclusion, namely that the STDP in basal dendrites seems to be controlled by parallel fiber information, is interesting and well argued via the NMDA mechanism. However, there is no ‘plasticity model’ to speak of. The argument for the presence of STDP is indirect. For instance, there is no model of the rate of synaptic change, and no discussion about the interaction between intrinsic and synaptic mechanisms. This would enrich the scope of the article, and I recommend including references and a concise discussion.

*We thank the reviewer for the appreciation and the comments that are helping to improve the paper. We agree on the STDP issue. Indeed, we aimed at addressing the effect of membrane potential on NMDA channels unblock and to predict, from it, the potential consequences for STDP. The lack of a generative STDP models (e.g. based on intracellular Ca-dependent mechanisms) in the paper was because STDP was yet experimentally undiscovered, so that we felt preliminary to anticipate any putative molecular processes. However, as suggested, we have now considered this issue in the discussion and added a modeling hypothesis in Supporting Information.*

The topic and problem are well introduced and the article goes straight to the point. The cerebellar loops are outlined and it is clear why it is important to look at “the fundamental issues that remain unexplored”. The article includes a substantially updated Golgi cell model which is an important piece of the cerebellar puzzle. The chosen ionic channels and their distributions along the cell are well justified and the need for the model is explained.

The inclusion of the model’s history is valuable, and the improvements from morphological and immunohistochemical data are compelling. Model optimization and validation are straight forward and well referenced. The model follows the electrophysiological and immunohistochemical data and is shown to respond well to different somatic current injections (eliciting sag, rebound bursting, phase-reset, etc).

*Thank you.*

STDP diagram is the standard one and is shown in Fig 9 how the changes in I_NMDA lead to LTD or LTP regions. These are referenced [13 and 31] but adding a short exposition of the conclusions of those articles will make the article more selfcontained.

*Indeed, this is now better commented in the discussion.*

It would also be very important to explain what are the relevant scales of change.

*The relative scale of changes along with hypothetical mechanisms are now discussed on the basis of known STDP at other brain synapses and of a simulation of STDP, based on the Shouval-Bear-Cooper model of plasticity, which is now reported in S9 figure.*

To my mind, the most important omission in the manuscript are the quantitative aspects of STDP. What is the expected maximum modulation of synaptic conductance via the mechanisms assumed? What is the rate of change? What is the Calcium level that switches from LTP and LTD?

*In order to get a quantitative insight into STDP, we have used the Shouval-Bear-Cooper model of plasticity in simulations using different numbers of mossy fiber synapse (1 or 11) and parallel fiber synapses (11, 31, 61, 81) (S9 figure), both stimulated with a 5spikes@100Hz burst. The Δ[Ca^2+^]_i._ generated by NMDA channels in the corresponding dendritic compartment reflects local Ca regulation, including removal due to diffusion and extrusion but not amplification by local calcium stores. This amplification, e.g. in the neighboring granule cell dendrites, is of about 3 times (84). Therefore, the Δ[Ca^2+^]_i._ caused by NMDA channel opening was multiplied by 3 times bringing Δ[Ca^2+^]_i._ around the STDP transition point (~0.75 μM). With a rate of change of 10, the model yields a classical STDP curve for the Golgi cell with a gain that depends on the amount of depolarization conveyed by the parallel fibers acting on apical dendrites. The STDP magnitude depended on the number of active parallel fibers and tended to plateau around ±40%. It should also be noted that the phase-dependence of the modelled STDP curve matches the pacemaking cycle of Golgi cells, that pulsate at theta rhythm. Therefore, the model supports the prediction that STDP would occur at mossy fiber synapses located on Golgi cell basal dendrites under parallel fiber control.*

Going forward it is important to understand the balance between LTP and LTD given expected statistics of synaptic inputs.

*We have now computed the input-output (I/O) space of the neuron using the combined activation of basal and apical dendrites by the corresponding input pathways. The simulations have been repeated using either 1 spike / input pathway or a short train of 5spikes@100Hz / input pathway. The color-coded maps of these two planes in the high-dimensional I/O space are now reported in S8 figure. Then, in 3 selected points of these I/O planes (corresponding to low, intermediate, and high neuronal output), we have unphased the stimuli reproducing the conditions for STDP. The results are reported in S9 figure. As guessed by the reviewer, the intensity of LTP and LTD changes depends on the position of the cell in the I/O space.*

For completeness of exposition, it would be relevant to include diagrams of the molecular mechanisms thought to underly bi-directional plasticity. While the mechanisms for LTP are clearly referenced, the detailed mechanisms for LTD are not as clear.

*Indeed, the mechanism of STDP used for simulations is now sketched in S9 figure along with the results of mechanistic simulations of STDP. The papers supporting the presence of the pathways and potential mechanisms involved are listed in supplemental material.*

— Suggestions for improvement:

- a simple sketch of the loops modeled would help readers that are not familiar with the cerebellum.

*done*

- more self-contained exposition (predicted by theory: what is the prediction and why?)

*corrected*

- discussion of molecular mechanisms involved

*Discussed with simulations shown in SM*

- discussion of the dynamic range and rate of STDP

*Discussed with simulations shown in SM*

Reviewer #1: Yes

Reviewer #2: **No:** Though the article says the models will be available in the HBP as a live article, no access has been provided for this reviewer (AFAICD).

*We added the link to modeldb.org with a couple of Golgi Cells model. The password is:* Golgi_2020
